# PReS-FINAL-2179: Efficacy and safety of adalimumab in pediatric patients with enthesitis related arthritis

**DOI:** 10.1186/1546-0096-11-S2-O14

**Published:** 2013-12-05

**Authors:** R Burgos-Vargas, S Tse, G Horneff, AL Pangan, K Unnebrink, JK Anderson

**Affiliations:** 1 Hospital General de Mexico, Universidad Nacional Autonoma de Mexico, Mexico City, Mexico; 2 University of Toronto, The Hospital for Sick Children, Toronto, Canada; 3 Asklepios Klinik Sankt Augustin, Sankt Augustin, Germany; 4 AbbVie Inc, North Chicago, IL, USA; 5 AbbVie Deutschland GmbH & Co. KG, Ludwigshafen, Germany

## Introduction

Enthesitis related arthritis (ERA) is a subcategory of juvenile idiopathic arthritis (JIA) which primarily affects peripheral joints and entheses but also can involve the sacroiliac joints and spine. It causes long-term effects on both physical and quality aspects of a child's life. Adalimumab (ADA) has been previously demonstrated to be effective in polyarticular JIA.

## Objectives

To evaluate the efficacy and safety of adalimumab compared to placebo in children and adolescents with ERA.

## Methods

This is a phase 3, multicenter, randomized, double-blind (DB) study in patients (pts) aged ≥6 to <18 years (yr) with ERA (ILAR criteria) with active disease not responsive to ≥1 nonsteroidal anti-inflammatory drug and ≥1 disease-modifying antirheumatic drug. Active disease was defined as ≥3 active joints (swelling or loss of motion + pain/tenderness) and enthesitis in ≥1 location. Pts were randomized 2:1 to receive blinded ADA (24 mg/m^2 ^BSA up to 40 mg every other week (wk) [eow]) or placebo (PBO) for 12 wks followed by open-label (OL) ADA eow up to 144 wks. The primary endpoint was% change from baseline (BL) in the number of active joints with arthritis (AJC) at wk 12. Secondary variables assessed included enthesitis count (EC), tender and swollen joint counts, and American College of Rheumatology (ACR) Pediatric (Pedi) 30/50/70 responses. Results are summarized through 52 wks of treatment. Safety was assessed in terms of adverse events (AE).

## Results

46 pts were randomized (31 to ADA, 15 to PBO). No pts discontinued during the DB period; however, 7 pts early escaped to OL ADA. Mean age was 12.9 ± 2.9 yrs. At BL, mean duration of ERA symptoms was 2.6 ± 2.3 yrs; mean AJC was 7.8 ± 6.6, and mean EC was 8.1 ± 8.4. The% change from BL at wk 12 in AJC was greater in the ADA group vs. PBO (-62.6 ± 59.5 vs -11.6 ± 100.5, *P *= 0.039). Most secondary variables showed numerically greater, but not statistically significant improvement at wk 12 in favor of ADA vs. PBO (Table). Treatment response was maintained with continued ADA therapy up to 52 wks (% change from BL at wk 52 in AJC, -88.7 ± 26.1). During the DB period AE incidence rates were similar [ADA/PBO (%)]: any AE (67.7/53.3), serious AE (3.2/0, 1 pt in the ADA group [abdominal pain and headache]), and infectious AEs (29.0/20.0). Among pts who received at least 1 dose of ADA through wk 52, any AE, serious AEs, and infectious AEs were reported in 91.3%, 10.9%, and 76.1%, respectively. No deaths, TB, or malignancies were reported.

**Table 1:** 

At Week 12		ADA (N = 31)	PBO (N = 15)
Change from Baseline^a^ (mean ± SD)	# enthesitis sites (0-35) Tender joint count (0-72) Swollen joint count (0-68)	-4.4 ± 6.2 -7.9 ± 8.3 -3.5 ± 5.6	-2.7 ± 5.0 -4.5 ± 9.0 -2.4 ± 4.7

ACR Pedi Response^b^ (n, %)	ACR Pedi30 responder ACR Pedi50 responder ACR Pedi70 responder	21 (67.7) 20 (64.5) 16 (51.6)	10 (66.7) 7 (46.7) 4 (26.7)

^a^LOCF. ^b^NRI. SD, standard deviation.

## Conclusion

ADA reduced the signs and symptoms of ERA at wk 12 and efficacy was sustained up to 52 wks. The safety profile observed in pediatric patients with ERA was consistent with that observed in children aged ≥4- years treated for polyarticular JIA.

## Disclosure of interest

R. Burgos-Vargas Grant/Research Support from: AbbVie, Consultant for: AbbVie, BMS, Pfizer, Speakers Bureau: AbbVie, BMS, Janssen, Pfizer, Roche, S. Tse Consultant for: AbbVie, Pfizer, G. Horneff Grant/Research Support from: AbbVie, Pfizer, Roche, Speakers Bureau: AbbVie, Novartis, Pfizer, Roche, A. Pangan Shareholder of: AbbVie, Employee of: AbbVie, K. Unnebrink Shareholder of: AbbVie, Employee of: AbbVie, J. Anderson Shareholder of: AbbVie, Employee of: AbbVie.

